# Natural Foods from Plant Sources in Preventing Nontransmissible Diseases

**DOI:** 10.1155/2018/6120103

**Published:** 2018-09-27

**Authors:** Almir Gonçalves Wanderley, Randhir Singh Dahiya, Sérgio Faloni De Andrade

**Affiliations:** ^1^Universidade Federal de Pernambuco, Departamento de Fisiologia e Farmacologia, Recife, Brazil; ^2^Maharishi Markandeshwar University, Ambala, India; ^3^Núcleo de Investigações Químico-Farmacêuticas (NIQFAR), Universidade do Vale do Itajaí (UNIVALI), Rua Uruguai, 458, Centro, 88302-202 Itajaí, SC, Brazil

The nontransmissible diseases also known as noncommunicable diseases (NCDs) or chronic diseases are noninfectious health condition that cannot spread from person to person. Generally, these diseases have slow progression and long duration. In accordance with World Health Organization, there are four main types of NCDs: (1) cardiovascular diseases (like heart attacks and stroke), (2) cancer, (3) chronic respiratory diseases (such as chronic obstructed pulmonary disease and asthma), and (4) diabetes. These diseases are responsible for 63% of all annual deaths provoking the death of more than 36 million people. Currently, these diseases kill more than all communicable diseases, such as HIV, malaria, and tuberculosis diarrhea.

There is growing evidence that positively correlates the consumption of natural foods with the reduction/prevention of diseases, mainly noncommunicable diseases. Within this criteria, consumption of plants and their derivatives represents important options in prevention of these diseases. Considering these view points, special issue in ECAM has been published, in order to report contributions of several researchers in this area.

In the present issue, seven articles have been published which are briefly described below.

In one of the articles, B. B. N'guessan et al. investigated the effect of CellGevity®, on rat liver microsomal cytochrome P450 (CYP) enzymes. This preparation is a dietary supplement contained riboceine (D-ribose-L-cysteine) a GSH-precursor molecule, which is reported to effectively deliver cysteine into the cell and enhance GSH level. Besides, CellGevity® contains other constituents such as turmeric root extract (curcumin), resveratrol, aloe extract, milk thistle, quercetin, broccoli seed extract, alpha lipoic acid, grape seed extract, vitamin C, selenomethionine, cordyceps, and piperine. Moreover, antioxidant potential of this dietary supplement* in vitro* was also estimated. The results showed that CellGevity® dietary supplement possesses moderate antioxidant activity* in vitro* and possesses inhibitory effect on selected rat liver CYP enzymes, suggesting its potential interaction with drugs metabolized by CYP enzymes.

In another study, S. Rampogu et al. undertook the chemical analyses from fenugreek (*Trigonella foenum-graecum*) seeds and also evaluated the potential of their main compounds against type 2 diabetes and breast cancer using molecular docking and molecular dynamics simulation-based computational drug discovery methods. The main compounds identified have been galactomannan and 4-hydroxyisoleucine and computational analysis displayed that galactomannan is an interesting compound from fenugreek seeds with a docking score compared to established drugs, such as canagliflozin and anastrozole in binding simulations of therapeutics against type 2 diabetes and breast cancer, respectively. Of this mode, the authors concluded that galactomannan, derived from fenugreek seeds, is a candidate for further experiments considering its value as a possible drug to treat type 2 diabetes and breast cancer.

In some countries fenugreek is commonly recommended as a galactagogue to breastfeeding women in case of hypogalactia. Thus, R. Shawahna et al. have analyzed the use of fenugreek among lactating women, in order to achieve formal consensus among breastfeeding women and healthcare providers on which potential harms and benefits of using fenugreek need to be communicated and discussed during the clinical consultations. The study involved breastfeeding women and healthcare providers to achieve formal consensus on a list of 24 and 16 items related to potential harms and benefits of fenugreek consumption during lactation. It achieved consensus about 21 potential harms and 14 potential benefits of using fenugreek to enhance human milk supply that needs to be discussed with breastfeeding women during consultations. Moreover, the authors pointed out that further observational studies are needed to assess what is being discussed in daily consultations when herbal remedies are recommended as galactagogue agents.

J. A. Pereira-Freire et al. investigated the phytochemistry profile and antioxidant potential of* Mauritia flexuosa* (Arecaceae) fruits and determined the bioaccessibility of its phenolic compounds.* M. flexuosa* is a palm tree widely distributed in South America, especially in the Amazon region and Brazilian Cerrado. In the Brazilian food industry, the peel and endocarp are commonly discarded or underutilized. The results have shown higher values for phenols, flavonoids, carotenoids, tannins, and ascorbic acid in peels when compared to the pulps and endocarps. Moreover, phenolic compounds identified by HPLC have shown reduced bioaccessibility after* in vitro* simulated gastrointestinal digestion. All samples showed capacity to scavenger free radicals but peels presented higher scavenger action in all methods explored and also protected rat blood cells against lysis induced by peroxyl radicals. Based on results, authors highlighted the nutritional characteristics of these by-products for human or livestock which otherwise are commonly discarded or are used as feed for ruminant animals only.

Another contribution of this special issue was the work of the H. Hong et al., which assessed the effects of* Glehnia littoralis* (GLE) root hot water extract and its underlying mechanism on 3T3-L1 cell adipogenesis and in high fat diet-induced obese mice. The GLE dose-dependently inhibited 3T3-L1 adipocyte differentiation and intracellular lipid accumulation in differentiated adipocytes. Further, body weight gain and fat accumulation were significantly lower in the GLE-treated HFD mice than in the untreated HFD mice and treatment suppressed the expression of adipogenic genes such as peroxisome proliferator-activated receptor (PPAR) *γ*, CCAAT/enhancer-binding protein (C/EBP) *α*, fatty acid synthase (aP2), and fatty acid synthase (FAS). These results suggested that GLE inhibits adipocyte differentiation and intracellular lipid accumulation by downregulating the adipogenic gene expression both* in vitro* and* in vivo*.

A Nationwide Cohort study was carried out by W.-C. Chen et al. to evaluate the effect of* Salvia miltiorrhiza* (Danshen) in the treatment of urolithiasis. The authors described that usage of* S. miltiorrhiza* decreased the ratio of subsequent stone treatment after the first treatment in the study population; there was no increased bleeding risk due to long-term use of it. Therefore, they suggested this is a safe herb having a potential for calculus prevention.

Finally, the effect of resveratrol suspension on the immune function of piglets has been evaluated by Q. Fu et al. showing that the treatment with it provoked significant effects on the development, maturation, proliferation, and transformation of T lymphocytes. The activity appears related to regulation of the humoral immune responses by upregulating the release of IFN-*γ* and downregulating the release of TNF-*α*. Moreover, there was significant increase in antibody titers of the piglets after immunization using swine fever vaccine (CSFV) and foot-and-mouth disease vaccine (FMDV). These positive effects indicate that resveratrol could be considered as an adjuvant to enhance the body's immune response to vaccines, as well as dietary additive for animals in order to enhance humoral and cellular immunity.

Thus, with contributions from research groups from different countries, this special issue exhibited that natural products have great potential to be explored in the prevention and treatment of not transmissible diseases. The editors of the special issue would like to thank all authors which submitted their works and allowed the success of this special issue.

